# Clinical study on Yanghe decoction in improving neo-adjuvant chemotherapy efficacy and immune function of breast cancer patients

**DOI:** 10.1097/MD.0000000000029031

**Published:** 2022-03-11

**Authors:** Xinyue Zhang, Minhao Hu, Siyu Li, Shanyan Sha, Ruoyu Mao, Yu Liu, Qiong Li, Qing Lu, Weili Chen, Ying Zhang, Rong Wang, Huaijin Xu, Jieqiong Wang, Yu Qiao, Ziyi Chen, Huangan Wu, Yuncui Pan, Qian Wang, Shuhui Zhang, Fan Yang, Jianwei Li, Guangyu Liu, Xiaohong Xue, Yajie Ji

**Affiliations:** a Department of Breast Surgery, Yueyang Hospital of Integrated Chinese and Western Medicine, Shanghai University of Traditional Chinese Medicine, Shanghai, China; b Shanghai Research Institute of Acupuncture and Meridian, Shanghai University of Traditional Chinese Medicine, Shanghai, China; c Department of Pathology, Yueyang Hospital of Integrated Chinese and Western Medicine, Shanghai University of Traditional Chinese Medicine, Shanghai, China; d Department of Breast Surgery, Key Laboratory of Breast Cancer in Shanghai, Fudan University Shanghai Cancer Center, Shanghai, China.

**Keywords:** breast cancer, immune function, neoadjuvant chemotherapy, traditional Chinese medicine, tumor response, Yanghe decoction

## Abstract

**Introduction:**

Neoadjuvant chemotherapy (NAC) plays an important role in downgrading preoperative tumor size, providing information on regimen activity, and increases treatment efficacy in breast cancer patients. An increasing number of patients have sought Traditional Chinese Medicine (TCM) during NAC to relieve discomfort, regulate immune function, and improve survival. However, limited evidence is available on how concurrent TCM treatment combined with NAC affects tumor response. This study aims to assess the efficacy of Yanghe decoction, a classical warming Yang formula, on pathological complete response (pCR) and explore its mechanism via the phosphatidylinositol-3-kinase/ protein kinase B/nuclear factor kappa-B (PI3K/Akt/NF-κB) pathway-mediated immune-inflammation microenvironment.

**Methods:**

A single-center, randomized, placebo-controlled, double-blinded randomized control trial (RCT) was designed. This trial aims to recruit 128 participants with breast cancer scheduled to receive NAC in China. All participants will be randomly assigned (1:1) to the Neo-Yanghe group (Yanghe decoction plus NAC) or the control group (placebo plus NAC). The primary outcome will be evaluated by the proportion of participants achieving pCR. The secondary outcomes include the expression level of PI3K/Akt/NF-κB pathway-related proteins, the objective response rate, the time to response, serum level of immune-inflammatory indicators, quality of life, disease-free survival, and overall survival.

**Discussion:**

This study will be the first RCT to evaluate the efficacy of Yanghe decoction combined with NAC in treating breast cancer patients, and elucidate the antitumor mechanism via the PI3K/Akt/NF-κB pathway-mediated immune-inflammation microenvironment. If possible, Neo-Yanghe treatment pattern will be a better pharmacological intervention to manage breast cancer than chemotherapy alone. The results of the trial will provide research-based evidence for the development of integrated Chinese and Western medicine guidelines and expert consensus.

**Trial registration:** Chinese Clinical Trial Registry ChiCTR-INR-2000036943. Registered on September 28, 2020 (https://www.chictr.org.cn/hvshowproject.aspx?id=57141).

## 1 Introduction

Female breast cancer has surpassed lung cancer as the most commonly diagnosed cancer, with an estimated 2.3 million new cases (11.7%) in 2020.^[1]^ It is also the leading cause of cancer-related death in Chinese women, with an incidence rate of 19.2% and a mortality rate of 9.1%.^[2]^ Chemotherapy plays a pivotal role in the comprehensive treatment of breast cancer, and neoadjuvant chemotherapy (NAC) has undergone considerable development. NAC is mainly utilized to preoperatively downgrade the size of the tumor, control disease, obtain prognostic information, and possibly improve survival.^[3]^ According to the National Comprehensive Cancer Network (NCCN) guideline, NAC has been accepted as standard care for preoperative patients with stage IIA-IIIC disease, especially those with human epidermal growth factor receptor 2 (HER2)-positive breast cancer and triple-negative breast cancer (TNBC).^[4]^ Pathological complete response (pCR), as a key indicator, is correlated with the efficacy of NAC and patient outcomes. Recently, studies have indicated that the overall efficiency of NAC is ∼60% to 90%, of which ∼30% to 60% of patients achieving pCR.^[5]^ According to the Chinese expert consensus on NAC,^[6]^ anthracyclines and taxus are recommended regimens for NAC treatment of breast cancer.

An inflammatory immune microenvironment is one of the hallmarks of cancer.^[7]^ Chronic inflammation is mediated through a variety of cytokines and hormones, which also contribute to breast cancer progression, metastasis, and therapy resistance in various ways.^[8]^ The phosphatidylinositol-3-kinase/protein kinase B (PI3K/Akt) signaling pathway plays a central role and is one of the most commonly altered pathways driving breast cancer cell growth, survival, and motility.^[9,10]^ Abnormal activation of the PI3K/Akt pathway is involved in the process of tumor infiltration and development.^[11]^ Nuclear factor kappa-B (NF-κB) is a downstream nuclear transcription factor of the PI3K/Akt signaling pathway. When NF-κB is activated, it can promote breast cancer development and contribute to the production of proinflammatory cytokines such as interleukins (ILs), vascular endothelial growth factor (VEGF), and tumor necrosis factor (TNF).^[12]^ Previous studies^[10,13–15]^ have revealed that dysregulation of the PI3K/Akt/NF-κB signaling pathway leads to the progression of breast cancer and is closely associated with tumor inflammation.

Chinese herbal formulas have become well known as a complementary therapy for their important role in the prevention and treatment of breast cancer. They can reduce toxicity or increase sensitivity to conventional chemotherapy and further regulate immune function. Our previous studies^[16–20]^ revealed that Chinese herbal formulas can inhibit distant metastasis of breast cancer, reduce chemotherapy-induced bone marrow suppression, regulate the Traditional Chinese Medicine (TCM) constitution, and attenuate endocrine therapy resistance. The warming Yang method in TCM is considered an important prophylactic and therapeutic treatment for breast cancer.^[21]^ The representative formula of Yanghe decoction can inhibit proliferation, reduce metastasis and induce the apoptosis of breast cancer cells, and its mechanism may be related to its inhibition of the activation of PI3K/Akt/NF-κB signaling pathway.^[22–26]^

However, there are limited data on the efficacy of Yanghe decoction combined with NAC for breast cancer patients. The purpose of this study was to evaluate the efficacy of additional Yanghe decoction on tumor response and immune function of breast cancer patients who received NAC.

## 2 Methods

### 2.1 Design and setting

**Figure 1:**
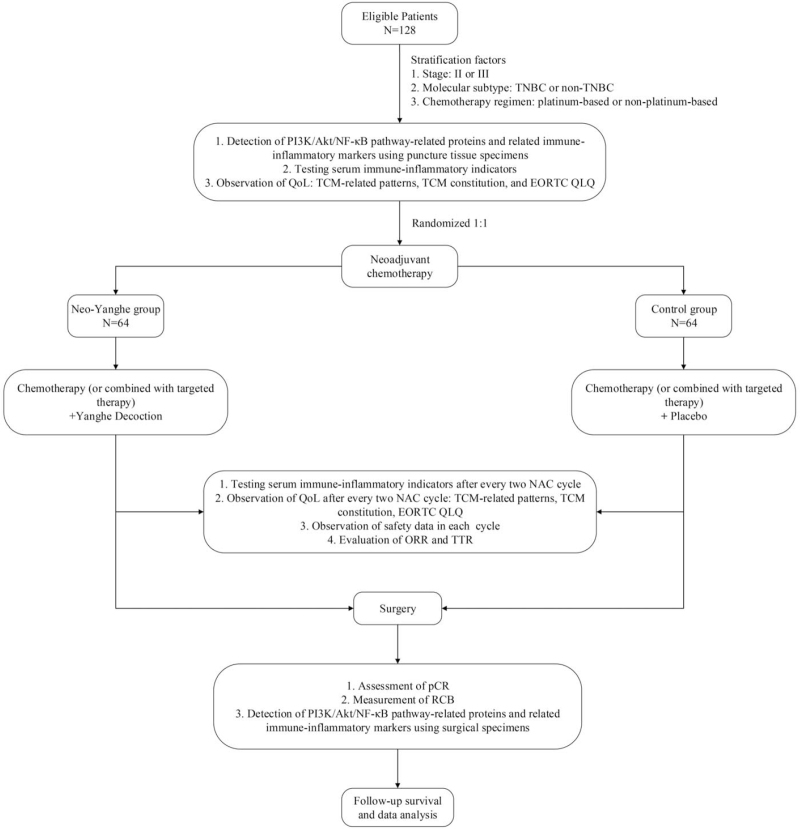
Overview of the study flow. EORTC QLQ = European Organization for Research and Treatment of Cancer, ORR = Objective response rate, pCR = Pathological complete response, QoL = Quality-of-life, RCB = Residual cancer burden, TCM = Traditional Chinese Medicine, TNBC = Triple-negative breast cancer, TTR = Time to response.

This study will be conducted in Yueyang Hospital of Integrated Chinese and Western Medicine, Shanghai University of Traditional Chinese Medicine. This study is a single-center, randomized, placebo-controlled, double-blinded clinical trial. Participants will be stratified into groups by randomized grouping. The factors for stratification include stage (II or III), molecular subtype (TNBC or non-TNBC) and chemotherapy regimen (platinum-based or non-platinum-based). As shown in Figure 1, 128 eligible participants will be randomized to the Neo-Yanghe group (Yanghe decoction plus NAC) or the control group (placebo plus NAC) in a 1:1 ratio. After standard treatment, all participants will receive surgery and be followed up. The final protocol (version 1.2) was finalized on March 23, 2021.

Ethical approval was received from the Institutional Review Board of the Yueyang Hospital of Integrated Chinese and Western Medicine, Shanghai University of TCM (No.: 2020-094). Written informed consent to participate will be obtained from all participants through their responsible physicians. Tumor tissue and serum samples will be collected. Informed consent will be obtained from all participants for biological specimen collection and use.

### 2.2 Participants

Participants who were initially diagnosed with breast cancer in the hospital between January 1, 2021 and December 31, 2023, will be enrolled.

#### 2.2.1 Inclusion criteria

1. Female.
2. Age 18 to 70 years.
3. Primary invasive breast carcinoma confirmed pathologically by biopsy.
4. Measurable tumor lesions (at least one) prior to treatment.
5. Clinical stage II to III and eligible to receive NAC.
6. No previous treatment for breast cancer.
7. Eastern Cooperative Oncology Group Performance Status (ECOG PS) ≤ 2.
8. Laboratory requirements: results of blood routine test, hepatic function test and renal function test are eligible for chemotherapy.
9. Signed informed consent form.

For the inclusion criteria, clinical diagnoses were made in accordance with the Tumor of the Breast (2nd Edition) edited by Zhimin Shao. Pathological diagnoses and molecular subtyping were performed with reference to the WHO Classification of Tumours of the Breast (5th Edition, 2019). Staging was based on clinical examination and pathological results according to the American Joint Committee on Cancer (AJCC) (8th edition) staging criteria.

#### 2.2.2 Exclusion criteria

1. Pregnant (serum β-HCG test) or lactating women.
2. Participants with proven distant metastases from breast cancer.
3. Participants with severe impairment of cardiac, hepatic or renal function.
4. Participants with other neoplastic disease.
5. Known hypersensitivity reaction to the drug or its excipients.
6. Participants with other conditions or diseases that, in the opinion of the investigator, may pose a significant risk to the patient or confound the results of the study.

### 2.3 Removal, dropout, and discontinuation criteria

1. Poor compliance and inability to complete the entire trial in strict compliance with the clinical protocol and on time.
2. Unwillingness to continue the clinical trial and requests to the supervising physician to withdraw.
3. Failure to start the study drug for various reasons after signing the informed consent form.
4. Statistical analysis of the data prior to the discussion with the statistician and principal investigator to determine exclusion.
5. Discontinuation of chemotherapy in the event of disease progression, death or unacceptable adverse reactions (AEs), followed by discontinuation of the trial drug and withdrawal from the trial.

### 2.4 Interventions

The control group is treated routinely without Yanghe decoction. The placebo, which is made of excipients and flavoring agents, is used as a comparator. The packaging, shape, weight, odor, and color of the placebo are identical to those of Yanghe decoction. The placebo was manufactured by the China Pharmaceutical Group Jiangyin Tianjiang Pharmaceutical Co, Ltd and was stored and provided to the participants by the Central Pharmacy of Yueyang Hospital.

#### 2.4.1 The Yanghe decoction

The setting and procedure for both groups are the same, except for the Neo-Yanghe group taking Yanghe decoction. The Yanghe decoction consists of Radix Rehmanniae Praeparata, Cornus cervi, Cinnamomum Cassia, Brassica alba Boiss, Rhizoma Zingiberis Preparata, and honey-processed Ephedra. These medicinal materials are boiled, concentrated and dried into formulated granules. When orally administered, 50 mL of boiling water is added and stirred until the granules are basically dissolved. Then, the appropriate amount of warm water is added for dilution the granule precipitate can be obtained. From the 7th day of each cycle of NAC to the first day before the next cycle of NAC (avoiding acute gastrointestinal reactions), Yanghe decoction granules or placebo are to be given to the Neo-Yanghe group or control group twice a day (1 bag each time), respectively. The Yanghe decoction granules were made by the China pharmaceutical group Jiangyin Tianjiang Pharmaceutical Co, Ltd and were stored and provided to the participants by the Central Pharmacy of Yueyang Hospital.

#### 2.4.2 Rationale of the Yanghe decoction

The representative warming Yang formula of Yanghe decoction was first recorded in the Life-saving Manual of Diagnosis and Treatment of External Diseases (Wai Ke Zheng Zhi Quan Sheng Ji) written by Hongxu Wang during the Qing Dynasty. The classical formula was designed to treat dorsal furuncles. The treatment of dorsal furuncle with Yanghe decoction is like the sun shining and the gloom dispersing, hence, it was termed “Yanghe.” According to TCM theory, this formula can warm Yang and nourish blood, resolve phlegm and unblock collaterals, replenish essence-Qi and support Yang-Qi, transform cold and restore channels. In summary, it treats the fundamental cause of disease by warming Yang.

#### 2.4.3 Chemotherapy regimen

All eligible participants will receive 6 to 8 cycles of NAC with the standard regimens recommended by the Guidelines of the Chinese Society of Clinical Oncology (CSCO) on the Diagnosis and Treatment of Breast Cancer (2020 version).^[27]^ The investigator will decide which regimens are suitable for the participants according to their pathology reports and systemic condition. Choices of NAC regimens include epirubicin plus cyclophosphamide (EC) × 4 times followed by docetaxel, with or without trastuzumab plus pertuzumab [T(HP)]  × 4 times; docetaxel, carboplatin, trastuzumab plus pertuzumab (TCbHP) × 6 times; docetaxel, doxorubicin plus cyclophosphamide (TAC) × 6 times; Paclitaxel, carboplatin, with or without trastuzumab plus pertuzumab [PCb(HP)] × 6 times.

### 2.5 Criteria for discontinuing or modifying allocated interventions

Participants may discontinue treatment at any time for any reason or at the discretion of the investigator in the event that an AE occurs. In addition, the investigator or sponsor may terminate a participant's treatment if they find the participant is unfit for treatment, violates the study protocol, or for administrative and/or other safety reasons.

### 2.6 Strategies to improve adherence to interventions

To improve adherence, the Central Pharmacy of Yueyang Hospital will prepare the granules and freely provide them to participants after each cycle of NAC. With the help of the WeChat management platform (ID: yanyueesf), the participants are instructed to take the granules twice a day. Linkdoc Technology Co., Ltd. will be responsible for telephone follow-up.

### 2.7 Outcomes

#### 2.7.1 Primary outcomes

The pCR rate will be compared between the Neo-Yanghe group and the control group.

#### 2.7.2 Secondary outcomes

1. Detection of PI3K/Akt/NF-κB pathway-related proteins and related immune-inflammatory markers.
2. Measurement of residual cancer burden (RCB).
3. Evaluation of the objective response rate (ORR) and time to response (TTR).
4. Testing of serum immune-inflammatory indicators.
5. Observation of safety data and the quality-of-life (QoL), including TCM-related patterns, TCM constitutions, and and European Organization for Research and Treatment of Cancer Quality of Life Questionnare (EORTC QLQ).
6. Determination of disease-free survival (DFS) and overall survival (OS).

### 2.8 Sample size

Comparisons between the Neo-Yanghe group and the control group are made in groups with 1:1 enrollment using the two-sided alternative hypothesis, with α (test level) = 0.05 and power 1-β (test efficacy) = 0.80. The pCR rate for breast cancer patients who receive NAC with the TAC regimen is approximately 30%.^[28]^ Our preliminary trial suggested that the pCR rate of additional Yanghe decoction was expected to be 55%, with a pCR of approximately 65% for the TNBC subtype and 45% for the non-TNBC subtype. The *Z*-test normal approximation method was used for estimation, Fisher's exact probability test was approximated, and the required total sample size was 116 cases, with 58 cases in each group. It was planned to recruit 128 participants, assuming a drop-out rate of 10%. Final pCR will be assessed at the time of the terminal analysis, with a data cutoff date of December 31, 2023.

### 2.9 Recruitment

The first participant enrolled on March 17, 2021. Up to September 14, 2021, a total of 16 participants were screened, and 10 participants were enrolled, 3 of whom completed all NAC cycles and underwent surgery. At the time of submission, recruitment is still ongoing.

### 2.10 Randomization and allocation concealment

Participants will be randomly assigned to the Neo-Yanghe group and the control group with a 1:1 allocation ratio. The stratification factors included stage (II or III), molecular subtype (TNBC or non-TNBC) and chemotherapy regimen (platinum-based or non-platinum-based). When an eligible participant is enrolled, the research assistant will register the detailed information of the participant, including birthday, full name and initials, and stratification factors. After uploading the participants’ information to the randomization website (http://crk.sdwgem.com/diaocha/manage/login.php), the website automatically generates the participants’ ID numbers and grouping results.

### 2.11 Blinding

Yanghe decoction granule and placebo granule are made the same color, taste, etc, and used the same packaging to maintain blinded status. Participants, investigators, and care providers in the treatment or outcome evaluation will be blinded.

For reasons of participant safety, the investigator may unblind participants on an emergency basis in the event of a medical emergency where the investigator needs to know the test drug being administered to the participants. Emergency unblinding must be performed by designated staff with delegated authority. Prior to unblinding the trial drug, the appropriate sponsor staff must be notified, and approval must be obtained from the principal investigator before subject-specific grouping information can be obtained. The investigator is required to document the time of the unblinding, the location of the unblinding, and the reason for the unblinding.

### 2.12 Data collection and management

Before the study began, relevant investigators and research staff were trained, and security measures were implemented for the equipment used, such as computers and data collection systems.

Data entry for the electronic case report form (eCRF), which is designed by Beijing Huajing Technology Co, Ltd, should be completed as soon as possible after the visit and kept up to date. To avoid differences in outcome assessment by different assessors, it is recommended that the same person complete the baseline and all subsequent efficacy and safety assessments for the same participant. Investigators are required to review the data to ensure the accuracy and correctness of all data entered into the eCRF. The time schedule of enrollment, interventions, and assessments are shown in Table 1.

**Table 1:** The time schedule of enrollment, interventions, and assessments.

		NAC treatment period										
Items	Baseline	1st cycle	2nd cycle	3rd cycle	4th cycle	5th cycle	6th cycle	7th cycle	8th cycle	Surgery	Follow-up	Protocol deviation
Visit date	√	√	√	√	√	√	√	√	√	√	√	
Informed consent	√											
Randomization	√											
Menopausal status	√											
Vital signs	√									√		
ECOG/KPS PS	√									√		
Family history of breast cancer	√											
Other medical history	√											
Blood routine tests	√	√	√	√	√	√	√	√	√			
Blood biochemistry tests	√	√	√	√	√	√	√	√	√			
Serum immune-inflammatory indicators tests	√		√		√		√		√			
Auxiliary examinations	√		√		√		√		√			
Breast Piercing pathology	√											
Lymph node piercing (if necessary)	√											
Expression level of PI3K/Akt/NF-κB pathway related proteins and related immune-inflammatory markers	√									√		
Clinical diagnosis	√									√		
Stratification factors	√											
Target lesions	√		√		√		√		√	√		
Chemotherapy regimen	√											
EORTC QLQ-C30	√		√		√		√		√			
EORTC QLQ-BR23	√		√		√		√		√			
TCM-related pattern	√		√		√		√		√			
TCM constitution	√		√		√		√		√			
Adverse Event		√	√	√	√	√	√	√	√			
Surgery information										√		
Pathological complete response assessment										√		
RCB assessment										√		
ORR and TTR									√	√		
Post-surgery treatment, such as radiotherapy and endocrine therapy, etc.											√	
DFS and OS											√	
Protocol deviation record form												√

DFS = disease free survival, ECOG PS = Eastern Cooperative Oncology Group Performance Status, EORTC QLQ = European Organization for Research and Treatment of Cancer-Quality of Life Questionnare, KPS = Karnofsky Performance Status, ORR = objective response rate, OS = overall survival, RCB = residual cancer burden, TCM = traditional Chinese medicine, TTR = time to response.

The frequency and plans for auditing trial conduct are reviewed every 3 months. Auditing trial conduct will be performed by a team independent from the investigators and the sponsor.

### 2.13 Outcome assessment

#### 2.13.1 Primary outcome assessment

Tumor pCR will be evaluated by the Independent Review Committee. pCR included breast pathological complete response (bpCR), total pathological complete response (tpCR) and near pathological complete response (near-pCR). bpCR was defined as the absence of residual invasive cancer cells in the breast but allowed the presence of carcinoma in situ in the breast and any residual tumor in lymph nodes, that is, ypT0/is ypN0/+.

tpCR was defined as no invasive or noninvasive residual cancer cells in the breast and lymph nodes, that is, ypT0ypN0. Near-pCR was defined as only a few scattered residual cancer cells remaining in the breast or the size of the remaining tumor being <0.5 cm.

#### 2.13.2 Secondary outcomes assessment

1. Detection of PI3K/Akt/NF-κB pathway-related proteins and related immune-inflammatory markers: Specimens will be collected before the start of NAC and post-NAC surgery. Western blotting and immunohistochemistry (IHC) will be used to test (i) the expression levels of PI3K/Akt/NF-κB pathway-related proteins, including PI3K, phosphor (p)-PI3K, Akt, p-Akt, NF-κB, p-NF-κB, inhibitor of NF-κB (IκB), and p-IκB, and (ii) the levels of immune-inflammatory markers, including COP9 signalosome 5 (CSN5), programmed cell death-1 (PD-1), programmed cell death ligand-1 (PD-L1), matrix metalloproteinase-9 (MMP-9), TNF-α, and VEGF. Furthermore, we will explore the correlation between the expression of PI3K/Akt/NF-κB pathway-related proteins and pCR. The above items will be carried out by the Department of Pathology of Yueyang Hospital.
2. Measurement of RCB: Surgical specimens are analysed for response by measurement RCB. The assessment of RCB includes 6 parameters, the bi-dimensional diameters of the primary tumor bed in the resection specimen (mm∗mm), overall cancer cellularity, the proportion of the primary tumor bed that contains ductal carcinoma, number of positive lymph nodes and diameter of largest metastasis (mm).^[29]^ After entering the above parameters into the RCB calculator (www.mdanderson.org/breastcancer_RCB), we will obtain the result of RCB class and RCB score.
3. Evaluation of ORR and TTR: ORR was defined as the proportion of patients with tumor size reduction in a predefined amount and for a minimum time period, including complete response (CR) and partial response (PR). TTR defined as the time to CR or PR. ORR and TTR will be evaluated by imaging after NAC and by pathology after surgery.
  According to RECIST 1.1 and in combination with physical examination, ultrasound and MRI findings, CR was defined as the complete disappearance of all target lesions. PR was defined as at least a 30% decrease in the sum of the longest diameter of measurable lesions (target lesions).
4. Testing serum immune-inflammatory indicators: The levels of serum immune-inflammatory indicators, including lymphocyte subsets (CD3, CD4, CD8, CD4/8), interleukin series (IL-2R/6/8/10) and tumor necrosis factor (TNF α/β), will be measured at the 0th, 2nd, 4th, 6th, and 8th (if present) cycles of NAC. The above items will be carried out by the central laboratory of Yueyang Hospital.
5. Observation of QoL: To collect and document AEs, TCM-related patterns, TCM constitution, and EORTC QLQ at the 0th, 2nd, 4th, 6th, and 8th (if received) cycles of NAC.
  TCM-related patterns will be evaluated according to the “Guideline for Diagnosis and Treatment of Tumors in TCM Chinese (ZYYXH/T135-156-2008)” edited by the China Association of Chinese Medicine. The TCM constitution is based on the judgment criteria of the “Criteria of Classification and Judgment of TCM Constitution (ZYYXH/T157-2009)” recognized by the China Association of Chinese Medicine.
  The EORTC QLQ-C30 and EORTC QLQ-BR23 wil be applied to assess patients’ QoL. The QLQ-C30 is a scale developed by the EORTC to measure the QoL of cancer patients. The QLQ-BR23 is a QoL questionnaire designed for breast cancer patients that is suitable for evaluating all breast cancer patients regardless of the type of treatment.
  The investigators will collect and document QoL scores of patients at the 0th, 2nd, 4th, 6th, and 8th (if received) cycles of NAC.
6. Survival: Follow-up will be conducted to determine one-year, three-year and five-year DFS and OS.

#### 2.13.3 Safety evaluation

1. Baseline information: The initials of the participant, trial medication code, birthday, phone number, outpatient or inpatient status, trial start date, chief complaint, and current medical history will be recorded.
2. Demographic characteristics: Age, sex, height, weight, and body surface area will be obtained.
3. Vital signs: Body temperature, heart rate, respiratory rate, blood pressure, and ECOG performance status will be measured.
4. Laboratory test results: Routine blood tests, routine urine tests, eight liver function tests (glutamic acid, alkaline phosphatase, total bilirubin, gamma-glutamate transferase, etc), five kidney function tests (urea nitrogen, serum creatinine, uric acid, etc), fasting blood glucose, four fasting lipid tests (total cholesterol, triglyceride, high-density lipoprotein; low-density lipoprotein), and seven tumor markers (carcinoembryonic antigen, alpha-fetoprotein, carbohydrate antigen (CA)-153, CA125, CA50, CA199, CA724) will be assessed.
5. Electrocardiogram
6. Whole-body ultrasound: Hepatobiliary, pancreatic, spleen, kidney, thyroid, and cervical lymph nodes, uterine adnexa and ovaries, and cardiac ultrasound to exclude distant metastases.
7. Chest CT: CT will be performed to exclude pulmonary metastases and to observe whether they are accompanied by regional lymph nodes involved, such as axillary, internal, subclavian and supraclavicular lymph nodes, and to measure the shortest diameter (mm).
8. MRI of the head: MRI will be performed to exclude brain metastases.
9. Bone scintigraphy: Scintigraphy will be performed to exclude bone metastases.
10. Tumor evaluation: Clinical palpation of the breast mass and regional lymph nodes (axillary, supraclavicular, and subclavicular), the longest and shortest diameter of the tumor by Vernier calipers (mm); breast and axillary ultrasound, bilateral mammography, breast MRI, and the longest diameter of the breast mass (mm) will be measured and recorded.
11. AEs: A scale of 0 to 5 will be used to grade AEs according to the Common Terminology Criteria for Adverse Events (CTCAE v5.0).

### 2.14 Confidentiality

The participants’ information will be de-identified, and a unique code will be assigned to each participant. Only the investigator and the site staff have access to the link between the participant's assigned code and the participant's identity. The confidentiality of records that could identify participants within the database must be protected, respecting the privacy and confidentiality rules.

### 2.15 Statistical methods

All statistical analyses will be performed using SPSS version 22.0 (SPSS, Inc., Chicago, IL). In general, the statistical analysis will be two-sided with α = 0.05. A bilateral 95% confidence interval (95% CI) will be provided if possible. Statistical significance is defined as *P* < .05.

Overall, the participants’ baseline demographic and disease characteristics will be summarized by descriptive statistics for both the Neo-Yanghe group and the control group. Continuous variables will be expressed as the number of participants, mean, median, standard deviation, max, and min. Categorical and ordinal variables will be expressed as a frequency and percentage. Stratified analysis, Cox regression, or correlation analysis will be used to explore the potential impact factors for different endpoints.

For primary objectives, comparisons between groups will be made with the Wilcoxon rank sum test or chi-square test. For secondary objectives, blood and tissue test markers and QoL will be assessed by t-tests or nonparametric tests. Description of the cases in which AEs occurred, the incidence rates of AEs and SAEs, and the composition ratio of SAEs will be calculated. The survival curve will be plotted by the Kaplan–Meier method. The log-rank test will be used to compare the differences between groups.

The full analysis set (FAS) which comprises all enrolled participants will be used as the primary analysis data set for the efficacy evaluation. The per-protocol set (PPS) which includes only participants who completed the trial will be used as secondary analysis data set for the efficacy evaluation.

### 2.16 Dissemination plans

We plan to submit the trial results to a peer-reviewed international journal, aiming to communicate with peers and public groups.

## 3 Discussion

Breast cancer is the most commonly diagnosed malignancy and a leading cause of cancer-related death in women in China. Chemotherapy plays a fundamental role in the treatment of breast cancer, but it also causes many short- and long-term AEs and often decreases QoL. TCM has been widely accepted as a mainstream form of complementary and alternative therapy with beneficial effects for breast cancer patients in China. An increasing number of patients seek TCM during chemotherapy to relieve symptom discomfort and to strengthen the body's defenses.

A previous study revealed that Yanghe decoction inhibited the growth of MDA-MB-231 cell lines, downregulated NF-κB protein expression and inhibited IL-8 secretion.^[24]^ Our team also found the simplified formula “Yanghe Tongluo Drink” (Patent Application Number: 201911006384.7) had remarkable efficacy on plasma cell mastitis after minimally invasive surgery. In addition, the potential antitumor mechanisms of Yanghe decoction have been screened through network pharmacology. The results showed that Yanghe decoction may act through the PI3K/Akt pathway to treat breast cancer. Based on our team's previous findings,^[30–32]^ we proposed exploring the clinical efficacy and experimental mechanism of Yanghe decoction combined with NAC for breast cancer patients. To validate the usefulness of Yanghe decoction combined with NAC for women with breast cancer, there is a need for controlled studies with well-defined and clinically relevant endpoints and adequate statistical power. As a well-organized prospective study, we expect the results will facilitate the translation of TCM concepts into the modern clinical practice of cancer management.

There are a number of limitations that should be considered. First, participants are not undergoing puncture biopsy to collect tumor tissue samples during NAC. Thus, the differences in pathological changes before and during NAC and the expression of relevant pathway indicators will not be observed. Second, the gastrointestinal side effects of chemotherapy has resulted in the Yanghe decoction not being administered on time or a dose reduction for a few enrolled patients. Third, a few patients needed to complete questionnaires with the help of others because of literacy problems, which may result in bias.

## Author contributions

YJ, XZ, MH, and SL contributed to the conception and design. XZ, MH, SL, SS, RM, YL, QLI, QLU, WC, YZ, RW, HX, JW, YQ, ZC, and HW were responsible for collection and assembly of data. MH, XZ, SL, SS, and RM were responsible for recording and analyzing the data. YP, QW, and SZ processed tissue specimens and delivered the pathological reports. FY, JL, GL, and XX were responsible for recommendation participants. XZ and YJ contributed to draft and interpret the article. All authors revised and approved the final manuscript.

**Conceptualization:** Xinyue Zhang, Minhao Hu, Siyu Li, Yajie Ji.

**Data curation:** Xinyue Zhang, Minhao Hu, Siyu Li, Shanyan Sha, Ruoyu Mao, Yu Liu, Qiong Li, Qing Lu, Weili Chen, Ying Zhang, Rong Wang, Huaijin Xu, Jieqiong Wang, Yu Qiao, Ziyi Chen, Huangan Wu.

**Formal analysis:** Xinyue Zhang, Minhao Hu, Siyu Li, Shanyan Sha, Ruoyu Mao.

**Funding acquisition:** Xiaohong Xue, Yajie Ji.

**Investigation:** Fan Yang, Jianwei Li, Guangyu Liu, Xiaohong Xue.

**Methodology:** Xinyue Zhang, Minhao Hu, Siyu Li, Yuncui Pan, Qian Wang, Shuhui Zhang.

**Project administration:** Fan Yang, Jianwei Li, Guangyu Liu, Xiaohong Xue.

**Writing – original draft:** Xinyue Zhang, Yajie Ji.

**Writing – review & editing:** Xinyue Zhang, Minhao Hu, Siyu Li, Shanyan Sha, Ruoyu Mao, Yu Liu, Qiong Li, Qing Lu, Weili Chen, Ying Zhang, Rong Wang, Huaijin Xu, Jieqiong Wang, Yu Qiao, Ziyi Chen, Huangan Wu, Yuncui Pan, Qian Wang, Shuhui Zhang, Fan Yang, Jianwei Li, Guangyu Liu, Xiaohong Xue, Yajie Ji.
